# Measuring treatment burden in people with Type 2 Diabetes Mellitus (T2DM): a mixed-methods systematic review

**DOI:** 10.1186/s12875-024-02461-x

**Published:** 2024-06-10

**Authors:** Kai Lin, Mi Yao, Xinxin Ji, Rouyan Li, Lesley Andrew, Jacques Oosthuizen, Moira Sim, Yongsong Chen

**Affiliations:** 1 Family Medicine Centre, The First Affiliated Hospital of Shantou University Medical College, Shantou, 515000 China; 2 School of Medical and Health Sciences, Edith Cowan University, Perth, 6027 Australia; 3 General Practice, Peking University First Hospital, Beijing, 100034 China; 4 School of Nursing and Midwifery, Edith Cowan University, Perth, 6027 Australia; 5 Endocrinology Department, The First Affiliated Hospital of Shantou University Medical College, Shantou, 515000 China

**Keywords:** Diabetes mellitus type 2, Treatment burden, Patient reported outcome measurement, Instrument, Measurement properties, Primary care settings

## Abstract

**Background:**

Measuring treatment burden is important for the effective management of Type 2 Diabetes Mellitus (T2DM) care. The purpose of this systematic review was to identify the most robust approach for measuring treatment burden in people with T2DM based on existing evidence.

**Methods:**

Articles from seven databases were retrieved. Qualitative, quantitative, and mixed-methods studies examining treatment burden in adults with T2DM and/or reporting relevant experiences were included. A convergent segregated approach with a mixed-methods design of systematic review was employed, creating a measurement framework in a narrative review for consistent critical appraisal. The quality of included studies was assessed using the Joanna Briggs Institute tool. The measurement properties of the instruments were evaluated using the Consensus based Standards for selection of Health Measurement Instruments (COSMIN) checklist.

**Results:**

A total of 21,584 records were screened, and 26 articles were included, comprising 11 quantitative, 11 qualitative, and 4 mixed-methods studies. A thematic analysis of qualitative data extracted from the included articles summarised a measurement framework encompassing seven core and six associated measurements. The core measurements, including financial, medication, administrative, lifestyle, healthcare, time/travel, and medical information burdens, directly reflect the constructs pertinent to the treatment burden of T2DM. In contrast, the associated measurement themes do not directly reflect the burdens or are less substantiated by current evidence. The results of the COSMIN checklist evaluation demonstrated that the Patient Experience with Treatment and Self-management (PETS), Treatment Burden Questionnaire (TBQ), and Multimorbidity Treatment Burden Questionnaire (MTBQ) have robust instrument development processes. These three instruments, with the highest total counts combining the number of themes covered and "positive" ratings in COSMIN evaluation, were in the top tertile stratification, demonstrating superior applicability for measuring T2DM treatment burden.

**Conclusions:**

This systematic review provides evidence for the currently superior option of measuring treatment burden in people with T2DM. It also revealed that most current research was conducted in well-resourced institutions, potentially overlooking variability in under-resourced settings.

**Supplementary Information:**

The online version contains supplementary material available at 10.1186/s12875-024-02461-x.

## Introduction

Type 2 Diabetes Mellitus (T2DM) constitutes over 90% of diabetes cases globally [1]. Managing T2DM entails complex treatments, various treatment-associated activities, and dealing with multiple complications, all of which place a significant burden on patients in terms of workload and costs [2]. Patients adhering to recommended T2DM treatments often consume numerous daily medications, visit healthcare professionals frequently, and invest substantial finances and time into their treatment [3]. These activities can result in the commitment of significant personal resources and impose unreasonable demands on patients, thereby increasing the treatment burden [4].

The treatment burden is an identified outcome of healthcare for people with chronic diseases, affecting behavioural, cognitive, physical, and psychosocial health of the individual [5]. This burden may arise when healthcare professionals prioritise treatment outcomes with limited regard for patient acceptability and feasibility, alongside insufficient coordination among specialists focusing on their respective areas of expertise [6]. Measuring the treatment burden in individuals with T2DM necessitates the quantification of multiple dimensions, such as aspects of financial, social, and psychological, which, however, present challenges when using observation or traditional estimations [7]. Wee et al. revealed the scarcity of instruments targeting the specific treatment burden among individuals with T2DM [8]. Additionally, existing instruments for assessing treatment burden may face difficulties in synthesising research findings due to heterogeneity in their development and conceptual foundations [9].

An effective approach to measuring treatment burden is crucial for integrating this concept into clinical guidelines. This integration has the potential to enhance patient experiences and outcomes by alleviating treatment burden [3, 9]. Patient-reported outcome measures (PROMs) evaluate individuals' experiences with disease and healthcare services, providing valuable data on outcomes from a patient's perspective [10, 11]. The aim of this study is to identify the most robust approach for measuring treatment burden in people with T2DM based on existing evidence.

## Methods

This systematic review follows the Preferred Reporting Items for Systematic Reviews and Meta Analyses (PRISMA) guidelines [12]. A protocol was registered on the International Prospective Register of Systematic Reviews (CRD42022244190). A convergent segregated approach of mixed-methods was used in this systematic review, integrating narrative review to summarise a measurement framework and critical appraisal to determine the most robust PROMs for measuring treatment burden in people with T2DM [13, 14].

### Retrieval formulas

A preliminary coding manual was developed from a literature review that identified variations in the measurement of treatment burden [11, 15, 16]. This manual facilitated the identification of keywords and concepts in retrieval formulas (Supplementary file, STable 1).

### Eligibility criteria

Peer-reviewed publications from inception to April 2022 were searched in four English and three Chinese databases. Studies to be considered eligible for inclusion should: (1) target adult populations (18 years and older) undergoing treatment for T2DM, and (2) qualitatively or quantitatively examine treatment burden or experiences that align with the conceptual framework proposed by Sav et al. [11], offering insights into patients' perceptions or cognitions concerning T2DM treatment burden. For quantitative studies included, additional criteria were added: (1) quantify treatment burden or relevant experiences in the target population using PROMs, and (2) specify the number of samples with T2DM. Studies that lacked a clearly defined sample of individuals diagnosed with T2DM or where the reported outcome focused on disease burden, diabetes distress, and treatment satisfaction were excluded.

### Searching for literature

The search strategy was developed through group discussions (K.L., M.Y., X.J., L.A., J.O., and M.S.). Bibliographic databases (Embase, PubMed, APA PsycInfo, Cumulative Index of Nursing and Allied Health Literature (CINAHL), China National Knowledge Infrastructure (CNKI), Wanfang and China Biomedical Literature Database Web (CBMWeb)) were searched using predefined Boolean operators, without filters or language restrictions. The research team used Rayyan software (https://rayyan.ai/, accessed February, 2022) to facilitate literature screening [17]. Four reviewers (K.L., M.Y., X.J., and R.L.) participated in the screening process. The screening process consisted of two stages: title and abstract screening, followed by full-text reading. Articles were included in the next stage unless all four reviewers agreed to exclude them.

### Quality assessment

Four reviewers independently assessed the quality of included studies and PROMs used in the studies. The Joanna Briggs Institute (JBI) Critical Appraisal Tool was employed for quality assessment, comprising nine domains specific to prevalence studies in the case of quantitative research, and ten domains for qualitative studies [18]. Evaluation for the studies with grades of "Yes", "No" or "Unclear" encompassing domains of the research design, conduct, analysis, and findings. A study was classified as low quality if more than three domains were rated as "No", "Unclear", or a combination of both. In cases of disagreement, a third-party (L.A., J.O., Y.C., M.S.) was consulted to resolve the issue.

### Data extraction

In the narrative review, reported qualitative data, findings from qualitative studies, and item descriptions from PROMs were considered as qualified qualitative data for exploring T2DM treatment burden. During the critical appraisal, PROMs used to measure treatment burden in the included quantitative studies were also extracted, along with the reported findings. The data extraction process was conducted by four reviewers using a standardised, pilot-tested spreadsheet. This spreadsheet captured key characteristics of the included studies, such as study design, period, geographic location, sample size, participant information, data collection methods, and main findings. The methods of instrument development were recorded in detail. To ensure data accuracy, authors of selected articles were contacted via email to clarify any missing or ambiguous information, and their feedback was integrated into the data.

### Narrative review

Initially, the narrative review was employed to summarise existing qualitative evidence into a thematic construct, representing the measurement framework for T2DM treatment burden. The narrative review employed Boell’s hermeneutic approach to summarise, interpret, and synthesise qualitative evidence from current peer-reviewed literature [19, 20]. The initial literature review identified a conceptual scope of treatment burden [11, 15, 16]. The conceptual framework proposed by Sav et al. for measuring generic treatment burden guided both the identification of literature and the data synthesis [11].

The data analysis and synthesis process encompassed four stages: coding, sorting, synthesising, and theorising [21]. The reported findings, qualitative data, and PROM items from the included studies were considered valid for qualitative analysis. An inductive thematic analysis was initially applied to the extracted qualitative data [22, 23]. This analysis was independently conducted by four reviewers (K.L., M.Y., X.J., and R.L.), beginning with a comprehensive reading and re-reading of the articles. The thorough examination led to the extraction of interpretive content that was relevant to the treatment burden of T2DM. Each extracted piece of content was coded using terminology derived from the original literature and was entered into the MAXQDA Analytics Pro 2020 software by reviewers independently. The reviewers identified recurring concepts within the data, which facilitated the generation of thematic codes related to the treatment burden experienced by individuals with T2DM. These codes were then collaboratively discussed until a consensus was reached among the reviewers. Subsequently, these thematic codes were organised into subthemes and themes. The entire analytical process, including the reviewed qualitative data, generated codes, and thematic terms, was subjected to a rigorous review by a third-party team (L.A., J.O., Y.C., M.S.).

Additionally, a panel with patient and public involvement and engagement (PPIE) was convened, consisting of four patients and four medical professionals from China’s primary care. The panel members were recruited through a primary care setting by a researcher (K.L.). Two structured discussions and feedback sessions were conducted to review the measurement framework. A custom scale was used to collect feedback, assessing the feasibility, appropriateness, meaningfulness, and effectiveness of the framework in the second session [13]. Content Validity Index (CVI) was calculated to evaluate the consistency of feedback.

### Critical appraisal of PROMs

The measurement framework derived from the narrative review served as the conceptual foundation for the subsequent critical appraisal of systematically included PROMs. This framework facilitates an in-depth exploration of the dimensional coverage of the included PROMs for measuring T2DM treatment burden, ensuring that the evaluation of the instrument development is both comprehensive and evidence-based. The measurement properties of PROMs were evaluated using the Consensus based Standards for selection of Health Measurement Instruments (COSMIN) checklist, assessing development process, reliability, validity, and responsiveness of a PROM, divided into ten domains [24]. Each domain was rated as very good, adequate, doubtful, inadequate, or not applicable (NA), with the lowest item rating determining the domain's overall rating. The "positive" results [25] were defined as obtaining "very good" and "adequate" ratings, indicated with a green background in Table 4, reflecting that the evidence supporting the measurement properties was sufficient.

### Ethics Statement

Ethical approval was not required, as this review exclusively included previously published data. All the studies included in our review were published in international, peer-reviewed journals. The researchers assessed the ethical considerations and adherence to relevant regulations of all included publications.

## Results

### Study selection

**Fig. 1:**
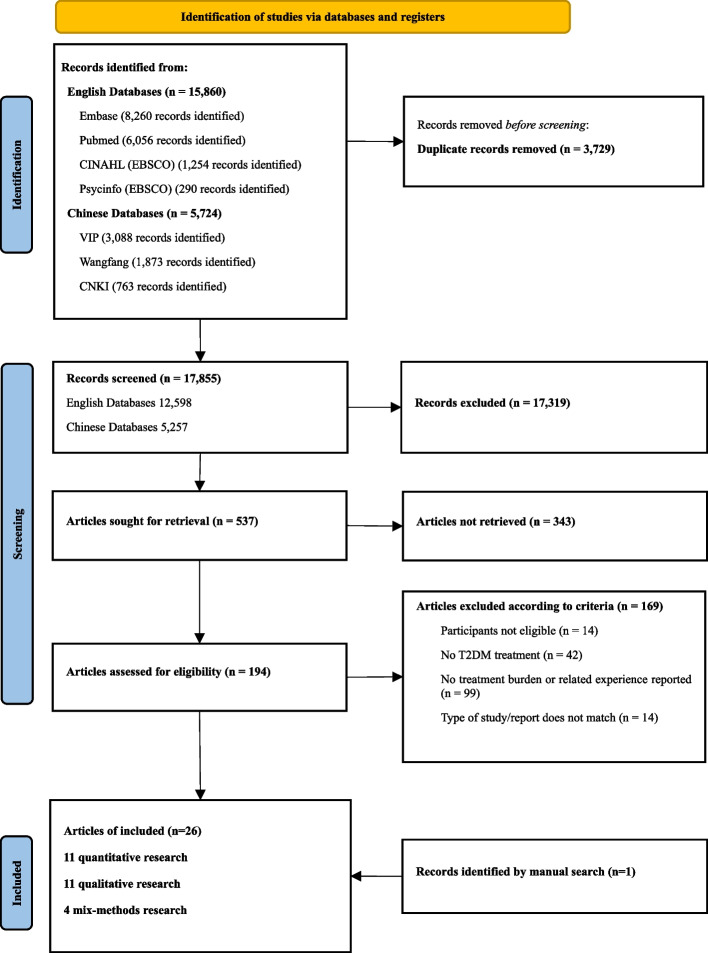
The PRISMA flow chart

Database searches identified 21,584 records for screening, and 194 records were retrieved for full-text review. A total of 26 articles were eligible for this review, including 11 quantitative studies, 11 qualitative studies, and 4 mixed-methods studies (Fig. 1). The narrative review included all 26 articles. Three of the four mixed-methods studies, despite their quantitative parts not meeting the inclusion criteria, provided valuable qualitative insights into multiple dimensions of T2DM treatment burden. The critical appraisal extracted PROMs from 12 quantitative studies, including 1 mixed-methods study; references related to the development of these PROMs were also reviewed by snowball searching but were not included in the search results, as they did not meet the inclusion criteria.

### Quality of included studies

In the quality assessment using the JBI tools (STable 2), the included quantitative studies had quality scores ranging from 4 to 9 (9 in total), whereas the qualitative studies had scores ranging from 4 to 8 (10 in total).

Common issues identified in quantitative studies were: (1) inconsistent and unverified measurement methods (8/12), (2) inadequate sample size (5/12) and (3) ambiguous descriptions for condition identification criteria (4/12); in qualitative studies were: (1) absence of cultural or theoretical frameworks (14/15), (2) misalignment between philosophical underpinnings and research methodology (13/15), (3) inadequate attention to the researcher's influence on the study and vice versa (12/15), and (4) insufficient representation of participant perspectives (12/15). No studies were excluded at this stage. Given the limited existing research in the field, all studies under consideration hold significant potential for contributing to the critical appraisal of instrument development, concept development, and hypothesis testing.

### Overview of included studies

The characteristics observed in the included studies are summarised in Table 1. Quantitative studies consisted of 12 cross-sectional questionnaire surveys with sample sizes ranging from 162 to 3,834 participants. Most studies were with people with T2DM (42%), while some included both people with Type 1 Diabetes Mellitus (T1DM) and T2DM (33%), and others involved people with T2DM from a larger non-communicable diseases (NCDs) population (25%). The majority of participants were adults aged 55–70 years (75%) and who received oral diabetic medications (58%). All qualitative studies were conducted with patients with T2DM; three studies also involved patients with T1DM or health care providers. Interviews were the most common data collection method (54%). Only 3 out of 26 studies were conducted in developing countries [26–28], with just one study from suburban areas in Ukraine [28], including participants from the low-resource environment [29].

**Table 1:** Summary of the included studies

First author (year)	Study method	Type of survey^a^	Period studied	Geographical location	Setting or service of recruitment	Participant information^b^	Sample size of T2DM	Mean Age (or range)	M/F%	Treatment^c^
**Quantitative studies (including 1 mixed-methods study)**										
Blüher, 2015 [57]	survey	DPROM	2009–2011	Germany	primary care setting	T2DM	3834	62.6 ± 10.8	44.78/55.22	oral
Brod, 2009 [47]	survey	DPROM	2002–2008	USA^d^	healthcare profiler	T1/T2	373	51 (18 ~ 80)	52.66/47.34	oral, inject
Gonz'alez-Saldivar, 2022 [26]	survey	DPROM	NA	Mexico	hospital, primary care setting	T1/T2	85	55.7 ± 12.9	37.30/62.70	NA
Han, 2022 [27]	survey	GPROM	2021	China	hospital	T2DM	300	68.16 ± 6.37	50.30/49.70	lifestyle, oral, inject
Herzig, 2019 [52]	survey	GPROM	NA	Switzerland	primary care setting	NCDs	277	72.9 ± 12.0	51.80/48.20	NA
Ishii, 2012 [55]	survey	DPROM	2010	Japan	outpatient	T1/T2	260	64.0 ± 11.6	40.10/59.90	oral, inject
Ishii, 2018 [56]	survey	DPROM	2016–2017	Japan	outpatient	T2DM	236	63.4 ± 11.9	39.80/60.20	oral, inject
Morris, 2021 [48]	survey	GPROM	2019	England	primary care setting	NCDs	248	75 ± 8.6	54.60/45.40	NA
Rogers, 2017 [50]	survey	GPROM	2014	USA	outpatient	T1/T2	120	63.9 (37 ~ 88)	59.00/41.00	NA
Sav, 2016 [49]	survey	GPROM	2013–2014	Australia	pharmacy	NCDs	171	57.28 ± 15.64	70.05/29.95	NA
Spencer-bonilla, 2021 [32]	survey	GPROM	2016–2017	USA	outpatient	T2DM	162	63.24 (54 ~ 71)	37.65/62.35	oral, inject
Vijan, 2005 [58]	survey	DPROM	2005	USA	mail	T2DM	1653	64 ± 11	NA	oral, inject
**Qualitative studies (including 4 mixed-methods study)**										
Bohlen, 2012 [38]	consultation video analysis	-	2006–2008	USA	outpatient	T2DM	46	62.33	NA	oral
Bustillos, 2020 [33]	interview	-	NA	USA	outpatient, home care	T2DM	31	≥ 65	26.00/74.00	NA
Cotugno, 2015 [43]	interview	-	2013	Australia	outpatient	T2DM	9	56	77.78/22.22	oral, inject
Crutzen, 2021 [34]	interview	-	2019	Netherlands	pharmacy	T2DM	16	66 (< 60 ~ > 80)	62.50/37.50	oral, inject
Dambha-miller, 2018 [44]	Open-end question	-	2002–2016	United Kingdom	primary care setting	T2DM	311	62.94	60.44/39.56	NA
Espinoza, 2020 [39]	focus group	-	2017	Chile	primary care setting	T2DM, clinician	30	35 ~ 75	NA	oral, inject
Fritschi, 2022 [40]	interview	-	2019–2020	USA	online	T2DM	8	68 ± 5.2	0/100	NA
Haider, 2021 [35]	consultation video analysis	-	2006–2008	USA	outpatient	T2DM	41	≥ 18	31.71/68.29	oral
Kristensen, 2018 [41]	interview	-	2015	Denmark	primary care setting	T2DM	13	59.2 (37 ~ 72)	38.00/62.00	oral, inject
Litterbach, 2020 [36]	Open-end question	-	2016	Australia	healthcare profiler	T1/T2	544	61 ± 9	50.00/50.00	oral, inject
Mandrik, 2013 [28]	focus group	-	NA	Ukraine	NA	T2DM	26	52.92 ± 8.00	53.85/46.15	oral, inject
Nair, 2007 [45]	interview	-	NA	Canada	primary care setting	T2DM	18	60 ± 13.3	55.60/44.40	oral, inject
Spencer-bonilla, 2021 [32]	interview	-	2016–2017	USA	outpatient	T2DM	17	63.24 (54 ~ 71)	NA	oral, inject
Tanenbaum, 2016 [37]	focus group	-	2014	USA	primary care setting	T2DM	32	55.86 ± 9.32	59.38/40.62	oral, inject
Vijan, 2005 [﻿^42^]	interview	-	NA	USA	primary care setting	T1/T2	6 groups	61	97.00/3.00	lifestyle, oral, inject

a *GPROM* Generic PROM, *DPROM* Diabetes specific PROM

b *T2DM* patients with T2DM, *T1/T2* patients with either T1DM or T2DM, *NCDs *patients with Noncommunicable diseases or multimorbidity; Sample size reporting is based on two scenarios: if the study solely involved T2DM cases, only the T2DM count was reported; if the study included T2DM cases and others, the T2DM count was provided along with the total sample size (Total) in parentheses

c *Lifestyle* lifestyle modification only, *Oral* oral medication, *Inject* injectable medication

d *USA*United States of America

### Result of narrative review

The literature screening process found that, while previous studies have used generic treatment burden scales to evaluate treatment burden in populations with NCDs, including patients with T2DM, there are unique concerns for people with T2DM [30, 31]. The PROMs currently used for measuring T2DM treatment burden lack a conceptual foundation with widely accepted consensus, making data synthesis challenging [7]. To critically appraise the extracted PROMs based on a specific and unified conceptual foundation, the narrative review of relevant qualitative studies was introduced.

The result of the thematic analysis represents a measurement framework for treatment burden in people with T2DM (Table 2). Seven themes had sufficient evidence to support their use as directly quantifiable indicators of the T2DM treatment burden, including financial [32–37], medication [35, 36, 38, 39], administrative [33, 35–38, 40, 41], lifestyle [33, 34, 36, 37, 39, 41, 42], healthcare [32, 35–39, 43, 44], time/travel [32, 33, 36, 43], and medical information [28, 34–36, 39, 43], and were categorised as core measurement themes. Sub-themes reflecting the antecedents [32, 36, 39, 41, 45] (patient characteristics, living with T2DM) and consequences [32, 33, 35–37, 41] (adherence to treatment, health and wellbeing and quality of life, interpersonal and social challenges) of the burden were encapsulated into associated measurement themes. Additionally, four novel themes related to T2DM treatment emerged, including health locus of control for T2DM treatment [33, 34, 36, 37, 41, 43], insulin or injection-related burden [36, 37], medication-related hypoglycaemia [28, 34], and glucose meters [37]. The final framework described themes and sub-themes, and also emphasised a circular interaction between core and associated measurement themes [11]. The PPIE panel provided feedback on the framework, with a CVI ranging from 0.81 to 1.00, indicating well acceptance (STable 3).

**Table 2:** The measurement framework of T2DM treatment burden

Themes	Category	Mentioned in studies (*n* = 15)	Mentioned in PROMs (*n* = 10)	Sub-themes
Financial	core measurements	6	4	Out-of-pocket expenses
				Costs associated with treatment
Medication	core measurements	4	8	Complexity of medication use
				Management of medications
				Drug dependence
				Side effect
Administrative	core measurements	7	7	Challenges of medical regimen
				Documentation and paperwork
				Arranging appointments
Lifestyle	core measurements	7	6	Challenges of health behaviours
				Change of nature behaviour
Healthcare	core measurements	8	3	Health care fragmentation
				Health care provider obstacles
				Difficulty navigating the health system
				Insurance or recourse use
Time/travel	core measurements	4	6	Transport difficulty
				Time spent
Medical information	core measurements	6	2	Cumbersome medical information
				Lack of effective sources of information
				Stigmatisation of treatment
Antecedents	associated measurements	5	1	Patient characteristics
				Living with T2DM
Consequences	associated measurements	6	7	Adherence to treatment
				Health and wellbeing and quality of life
				Interpersonal and social challenges
				Satisfaction with treatment
Health locus of control for T2DM treatment	associated measurements	6	4	
Insulin- or injection-related burden	associated measurements	2	2	
Medication-related Hypoglycaemia	associated measurements	2	2	
Glucose meters	associated measurements	1	1	

### Result of critical appraisal

In total, 10 PROMs were extracted from the included quantitative studies. Table 3 summarises these 10 instruments and shows the coverage of measurement themes for each within the measurement framework. The Patient Experience with Treatment and Self-management (PETS, 7/7), Treatment Burden Questionnaire (TBQ, 6/7), and Multimorbidity Treatment Burden Questionnaire (MTBQ, 7/7) covered a wide range of the core measurement themes. Notably, despite deficiencies in core measurement themes, the Diabetic Treatment Burden Questionnaire (DTBQ), Diabetes Therapy-Related QOL (DTR-QOL), Treatment Related Impact Measures: Diabetes and Diabetes Device (TRIM-D and TRIM-DD), and one of the Self-Made Questionnaires (SMQ-3) encompassed partial novel themes related to T2DM treatment. However, none of the included PROMs fully matched all the themes in this framework.

**Table 3:** Summary of the PROMs extracted

Instrument^a^	Category^b^	Number of domains	Number of items	Response options	Sample size	Age range	Treatment^c^	CORE measurements covered	ASSOCIATED measurements covered	Count of themes covered (CORE/ ASSOCIATED)
PETS [32, 50]	GPROM	10	48	5	120 ~ 162	63.24 ~ 63.90	Oral, Inject	Financial, Medication, Administrative, Lifestyle Healthcare, Time/travel, Medical information	Consequences	7/1
TBQ [27, 49, 52, 53]	GPROM	5	15	5	171 ~ 300	57.28 ~ 72.90	Lifestyle, Oral, Inject	Financial, Medication, Administrative, Lifestyle Healthcare, Time/travel	Antecedents, Consequences, Insulin- or injection-related burden, Glucose meters	6/3
MTBQ [48, 54]	GPROM	3	12	5	248	75.00 ± 8.60	NA	Financial, Medication, Administrative, Lifestyle Healthcare, Time/travel, Medical information	Consequences	7/1
DTBQ [56]	DPROM	3	18	7	236	63.40 ± 11.90	Oral, Inject	Medication, Lifestyle, Time/travel	Health locus of diabetes control, Medication-related Hypoglycaemia	3/2
DTR-QOL [55]	DPROM	4	29	7	260	64.00 ± 11.60	Oral, Inject	Lifestyle, Time/travel	Antecedents, Consequences, Health locus of diabetes control, Medication-related Hypoglycaemia	2/4
TRIMs [47]	DPROM	7	36	5	373	51 (18 ~ 80)	Oral, Inject	Medication, Administrative	Consequences, Health locus of diabetes control	2/2
SMQ-1 [58]	DPROM	NA	10	7	1653	64 ± 11	Oral, Inject	Medication, Administrative, Lifestyle	none	3/0
SMQ-2 [57]	DPROM	NA	6	4	3834	62.60 ± 10.80	Oral	Medication, Time/travel	Consequences	2/1
SMQ-3 [26]	DPROM	5	33	3	204	55.70 ± 12.90	NA	Financial, Medication, Administrative	Consequences, Health locus of diabetes control, Insulin- or injection-related burden	3/3
SMQ-4 [48]	GPROM	NA	1	11	248	75.00 ± 8.60	NA	NA	NA	NA

a *PETS* Patient Experience with Treatment and Self-management, *TBQ *Treatment Burden Questionnaire, *MTBQ* Multimorbidity Treatment Burden Questionnaire, *DTBQ* Diabetic Treatment Burden Questionnaire, *DTR-QoL* Diabetes Therapy-Related Quality of Life, *TRIMs* Treatment Related Impact Measures, *SMQ *self-made questionnaire

b *GPROM* Generic PROM, *DPROM* Diabetes specific PROM

c *Lifestyle* lifestyle modification only, *Oral* oral medication, *Inject* injectable medication

All extracted PROMs were evaluated for their measurement properties using the COSMIN checklist (Table 4). The results indicate that PETS, TBQ, and MTBQ are appropriate PROMs for measuring the T2DM treatment burden, with the strongest evidence on measurement properties. These PROMs demonstrated that the majority of their measurement properties received a "positive" rating, with robust reliability, and content and structural validity. Notably, all PROMs (10/10) were rated "negative" in criterion validity, attributed to the lack of testing against a consensus gold standard metric for treatment burden. Additionally, most PROMs (8/10) were rated "negative" in terms of responsiveness, due to their limited application in longitudinal studies.

**Table 4:** Result of critical appraisal (COSMIN checklist)

PROMs	Instrument development^a^	Content validity	Structural validity	Internal consistency	Cross-cultural validity	Reliability	Measurement error	Criterion validity	Hypothesis testing	Responsiveness
PETS	Adequate	Very good	Very good	Very good	Adequate	Very good	Adequate	Doubtful	Adequate	Doubtful
TBQ	Doubtful	Adequate	Very good	Very good	Very good	Very good	Adequate	Doubtful	Doubtful	Doubtful
MTBQ	Doubtful	Adequate	Adequate	Adequate	Adequate	Very good	Doubtful	Doubtful	Doubtful	Adequate
DTBQ	Inadequate	Doubtful	Doubtful	Adequate	NA	Adequate	NA	NA	Doubtful	NA
DTR-QOL	Inadequate	Doubtful	Doubtful	Adequate	NA	Adequate	NA	NA	Doubtful	NA
TRIMs	Inadequate	Doubtful	Doubtful	Very good	Adequate	Very good	Adequate	Doubtful	Doubtful	Doubtful
SMQ-1	Inadequate	Inadequate	Doubtful	Adequate	NA	Doubtful	Doubtful	NA	Inadequate	NA
SMQ-2	Inadequate	Inadequate	Inadequate	Adequate	Doubtful	Adequate	Inadequate	NA	Doubtful	NA
SMQ-3	Doubtful	Doubtful	Inadequate	Inadequate	NA	Inadequate	Adequate	NA	Doubtful	NA
SMQ-4	NA	NA	NA	NA	NA	NA	Doubtful	Doubtful	NA	Adequate

^a^ Very Good: Strong evidence supporting the measurement property. Adequate: Sufficient evidence supporting the measurement property. Doubtful: Some evidence present, but it is incomplete or of questionable quality. Inadequate: Insufficient evidence or poor-quality evidence. Not Applicable: No evidence available, often due to the nature of the instrument not being designed or not used to measure the property in question

## Discussion

### Call for a specific and unified measurement framework

A total of 10 PROMs were extracted from the systematic search and evaluated for their measurement properties. During the searching and screening stage, heterogeneity was found in the research objectives, instruments of the included quantitative surveys, and the measurement structures of these PROMs. While previous studies have used generic treatment burden scales to evaluate treatment burden in populations with NCDs, including patients with T2DM, there are unique concerns for people with T2DM [30, 31]. The PROMs currently used for measuring T2DM treatment burden lack a conceptual foundation with widely accepted consensus [7]. This has led to variations in the number of dimensions measured and the outcome paradigms utilised for measurement. This heterogeneity has been previously reported by Lesage et al., highlighting the challenges in conducting outcome data synthesis [9]. To address this, a convergent segregated mixed-methods approach was introduced [13, 14].

To critically appraise the extracted PROMs based on a specific and unified conceptual foundation, a narrative review of relevant qualitative studies was employed. Thematic analysis was conducted on qualitative data, including reported qualitative findings and item descriptions from PROMs. This analysis refined existing knowledge [11, 15, 16] to elucidate the concept of treatment burden, with a specific focus on T2DM care. The measurement framework in Table 2 indicates that seven themes were categorised as core measurement themes. These themes represent issues of T2DM treatment burden that can be directly reflected by PROMs, evident in previous qualitative studies and utilised in existing T2DM treatment burden measurements [46]. While the core measurement themes encompass significant components of treatment workload and patient burdens, the associated measurement themes primarily consist of components that do not directly reflect these burdens. The associated measurement themes typically reflect factors that influence or are influenced by the treatment burden in people with T2DM [27, 47–49].

Due to limited evidence, the review categorised the four emergent themes (Health locus of control for T2DM treatment, Insulin- or injection-related burden, Medication-related Hypoglycaemia, Glucose meters) identified in the narrative review as associated measurement themes. These themes relate to specific burdens in people with T2DM that are inadequately captured by existing generic patient-reported outcome measures. Feedback on these constructs was sought from the panel with PPIE for framework validation.

### Instrument selection

The selection of instrument impacts research methodology and the quality of findings [18]; the theoretical underpinnings and developmental principles of the selected instrument are pivotal to the validity of outcomes [46]. In our narrative review, the measurement framework was constructed to provide a conceptual foundation for the critical appraisal of the extracted PROMs measuring T2DM treatment burden. This framework, combined with the COSMIN checklist, was used for the critical appraisal of the included PROMs. The PROMs were stratified to facilitate a comparison according to the number of themes covered in the measurement framework (Table 3) and the number of "positive" ratings received in the COSMIN checklist (Table 4) by each instrument. The three PROMs with the highest total counts, combining the number of themes covered and "positive" ratings, were PETS, TBQ, and MTBQ. These PROMs, in the top tertile stratification, demonstrated superior applicability for measuring T2DM treatment burden.

PETS (Rogers, 2017) [32, 50] was identified as the most comprehensive among the included PROMs, capturing the majority of core measurements outlined in the framework and allowing for segregated score calculations [51]. PETS also partially addressed hypothesis testing in the T2DM population through exploratory analyses comparing mean subscale scores across groups with varying levels of glycaemic control [32]. It was the only one out of the ten PROMs (Table 4) received "positive" ratings in PROM development and hypothesis testing. However, the evidence supporting these domains of PETS is not strong enough. The PETS instrument was initially designed to measure the treatment burden in people with chronic diseases in general, not specifically for T2DM. PROM development and hypothesis testing related to the T2DM population were conducted in subsequent studies. The differences in disease-specific concerns may result in the omission of certain T2DM-specific issues on treatment burden, echoing the discussion in the last Sect. [9]. Furthermore, the validation of PETS primarily involved participants with higher education levels from well-resourced settings, suggesting potential limitations in applying PETS in under-resourced environments or developing countries [51]. Comparable to PETS, the TBQ (Tran, 2014) [27, 49, 52, 53] and MTBQ (Duncan, 2018) [48, 54] demonstrated similarly broad thematic coverage and substantial instrument validation. Nonetheless, these instruments also displayed deficiencies in their developmental and validation processes concerning T2DM-specific PROMs.

DTR-QoL [55] [55], TRIMs (Brod, 2009) [47], DTBQ (Ishii, 2018) [56], SMQ-3 (González-Saldivar, 2022) [26], and SMQ-2 (Blüher, 2015) [57], exhibited intermediate levels of thematic coverage and received moderate positive ratings on the COSMIN checklist. A predominant limitation for this group of PROMs is their circumscribed thematic scope, coupled with insufficient structural validation pertaining to T2DM treatment burden. SMQ-1 (Vijan, 2005) [58] and SMQ-4 (Morris, 2021) [48] demonstrated limited thematic coverage and an inadequate instrument development process. Being self-developed PROMs with insufficient validation, these instruments are not recommended for measuring treatment burden in people with T2DM.

### Limitations and inspiration

Given the preliminary search results in this review, which indicated that previous research on treatment burden and instrument development was primarily conducted in developed or well-resourced settings, three Chinese databases were also searched in addition to the commonly used medical databases. This aimed to include complementary sources from developing countries and low-resource settings. However, the result shows that only a limited of the included studies (11.5%, including two quantitative and one qualitative study) were conducted in developing countries or low-resource settings [26–28]. Moreover, the results of the JBI quality assessment show that all these studies had a high risk of bias. Further research with high-quality input from patients and healthcare professionals in low-resource settings is essential to create a specialised measurement paradigm that accurately represents the treatment burden in individuals with T2DM in such contexts.

Additionally, 8 out of 10 PROMs were rated "negative" in the domain of responsiveness. The majority of the included quantitative studies were cross-sectional surveys, which hindered the evaluation of the instruments' responsiveness. This reflects the lack of longitudinal studies in current treatment burden research, obstructing the refinement of measurements and causes existing instruments to fall short in assessing changes in treatment burden over time. On the other hand, the absence of a current "gold standard" for measuring treatment burden resulted in all evaluated PROMs (10/10) being rated "negative" in criterion validity. These limitations suggest a critical need for the development and validation of a consensus-based standard and the implementation of longitudinal studies to improve the accuracy and responsiveness of treatment burden assessments. Alternatively, developing instruments with a specific and unified framework will facilitate further measurement of T2DM treatment burden and synthesis of research outcomes.

Finally, this review evaluated PROMs solely based on their development process and measurement properties. In addition to these internal parameters, it is crucial to consider how the measured levels of treatment burden correlate with other healthcare indicators, such as blood glucose control or patients' experiences [59]. These correlations should also be taken into consideration when determining the suitability of a particular instrument for a specific context.

## Conclusions

Understanding treatment burden is essential to patient-centred care. This systematic review provides evidence for the currently superior options for measuring treatment burden in people with T2DM. The results indicate that PETS, TBQ, and MTBQ demonstrated their robust evidence for measuring T2DM treatment burden. However, as generic PROMs, clinicians should be aware of their limitations and consider the specific context when using these instruments, especially in developing countries or under-resourced settings.

### Supplementary Information

Supplementary Material 1.

## Data Availability

This systematic review utilised data from previously published studies, all of which are accessible through their respective journals and databases. The data supporting the conclusions of this review are available in the cited articles, and/or can be obtained from the corresponding authors upon reasonable request.
