# Supplementary material for: Measuring treatment burden in people with Type 2 Diabetes Mellitus (T2DM): a mixed-methods systematic review
Source: BMC Prim Care. 2024 Jun 10;25:206. doi: 10.1186/s12875-024-02461-x (PMC11165743; doi:10.1186/s12875-024-02461-x)
Supplement: Supplementary file 1 — Supplementary Material 1. [file 12875_2024_2461_MOESM1_ESM.docx]

**Supplementary file**

STable 1 Retrieval formulass:

Retrieval formula for English health-based databases:

| Embase |
| --- |
| #1 'type 2 diabetes'  #2 't2d*'  #3 'non insulin dependent diabetes mellitus'  #4 #1 OR #2 OR #3  #5 medication  #6 treatment  #7 intervention  #8 therap*  #9 #5 OR #6 OR #7 OR #8  #10 burden  #11 workload  #12 experience  #13 #10 OR #11 OR #12  #14 #9 AND #13  #15 questionnaire* OR survey* OR instrument* OR PRO* OR scale* OR tool* OR measur* OR assess* OR evaluat*  #16 #4 AND #14 AND #15 |

| Pubmed |
| --- |
| (((type 2 diabetes or T2D or T2DM) AND (medication or treatment or intervention or therap*)) AND (burden or workload or experience)) AND (questionnaire* or survey* or instrument* or pro* or scale* or tool* or measur* or assess* or evaluat*) |

| CINAHL, Psycinfo (EBSCO) | | |
| --- | --- | --- |
| S1 | type 2 diabetes mellitus | Apply related words; Apply equivalent subjects |
| S2 | diabetes mellitus | Apply related words; Apply equivalent subjects |
| S3 | diabet* | Apply related words; Apply equivalent subjects |
| S4 | MODY | Apply related words; Apply equivalent subjects |
| S5 | NIDDM | Apply related words; Apply equivalent subjects |
| S6 | T2DM | Apply related words; Apply equivalent subjects |
| S7 | T2D | Apply related words; Apply equivalent subjects |
| S8 | non insulin* depend* | Apply related words; Apply equivalent subjects |
| S9 | noninsulin* depend* | Apply related words; Apply equivalent subjects |
| S10 | noninsulindepend* | Apply related words; Apply equivalent subjects |
| S11 | non insulindepend* | Apply related words; Apply equivalent subjects |
| S12 | S1 or S2 or S3 or S4 or S5 or S6 or S7 or S8 or S9 or S10 or S11 | Apply related words; Apply equivalent subjects |
| S13 | burden of disease | Apply related words; Apply equivalent subjects |
| S14 | disease burden | Apply related words; Apply equivalent subjects |
| S15 | treatment burden | Apply related words; Apply equivalent subjects |
| S16 | burden of treatment | Apply related words; Apply equivalent subjects |
| S17 | medication burden | Apply related words; Apply equivalent subjects |
| S18 | burden of medication | Apply related words; Apply equivalent subjects |
| S19 | time burden | Apply related words; Apply equivalent subjects |
| S20 | travel burden | Apply related words; Apply equivalent subjects |
| S21 | cost burden | Apply related words; Apply equivalent subjects |
| S22 | treatment experiences | Apply related words; Apply equivalent subjects |
| S23 | workload | Apply related words; Apply equivalent subjects |
| S24 | S13 or S14 or S15 or S16 or S17 or S18 or S19 or S20 or S21 or S22 or S23 | Apply related words; Apply equivalent subjects |
| S25 | measur* | Apply related words; Apply equivalent subjects |
| S26 | measurement | Apply related words; Apply equivalent subjects |
| S27 | instrument | Apply related words; Apply equivalent subjects |
| S28 | scale | Apply related words; Apply equivalent subjects |
| S29 | test | Apply related words; Apply equivalent subjects |
| S30 | questionnaire | Apply related words; Apply equivalent subjects |
| S31 | pro* | Apply related words; Apply equivalent subjects |
| S32 | assess* | Apply related words; Apply equivalent subjects |
| S33 | inventory | Apply related words; Apply equivalent subjects |
| S34 | tool | Apply related words; Apply equivalent subjects |
| S35 | survey | Apply related words; Apply equivalent subjects |
| S36 | S25 or S26 or S27 or S28 or S29 or S30 or S31 or S32 or S33 or S34 or S35 | Apply related words; Apply equivalent subjects |
| S37 | S12 AND S24 AND S36 | Apply related words; Apply equivalent subjects |

Retrieval formula for Chinese health-based databases (Translated):

| Wanfang |
| --- |
| Title or keywords: (("Type 2 diabetes" or "T2DM" or "T2D*" or "diabetes") and ("medication" or "intervention" or "treatment" or "therapy" ) and ("burden" or "workload" or "cost" or "experience") and ("measurement" or "evaluation" or "assessment" or "scale" or "questionnaire" or "pro")) or Abstract: (("Type 2 diabetes" or "T2DM" or "T2D*" or "diabetes") and ("medication" or "intervention" or "treatment" or "therapy") and ("burden" or "workload" or "expense" or "experience") and ("measurement" or "evaluation" or "assessment" or "scale" or "questionnaire")) |
| CNKI |
| ((TI=("Type 2 Diabetes" + "T2DM" + "T2D*" + "Diabetes") ) OR (AB=("Type 2 diabetes mellitus" + "T2DM" + "T2D*" + "diabetes mellitus "))) AND ((TI=("medication" + "intervention" + "treatment" + " therapy")) OR (AB "drug" + "intervention" + "treatment" + " therapy"))) AND ((TI=( "burden" + "workload" + "cost" + "experience ")) OR (AB=( "burden" + "workload" + "cost" + "experience "))) AND ((TI=("Measurement" + "Evaluation" + "Assessment" + "Scale " + "Questionnaire"+ "Pro")) OR (AB=("Measure" + "Evaluate" + "Assess " + "scale" + "questionnaire"+ "Pro"))) |
| CBMWeb (cqvip) |
| ((M=(("Type 2 diabetes" or "T2DM" or "T2D*" or "diabetes") AND ("medication" or "intervention" or "treatment" or " therapy") AND ("burden" or "workload" or "cost" or "experience") AND ("measurement" or "evaluation" or "assessment" or "scale" or "questionnaire" or "Pro")) OR ( R=((("Type 2 diabetes" or "T2DM" or "T2D*" or "diabetes") AND ("medication" or "intervention" or "treatment" or " therapy") AND ("burden" or "workload" or "cost" or "experience") AND ("measurement" or "evaluation" or "assessment" or "scale" or "questionnaire" or "Pro"))) |

STable 2 Result of JBI checklist

| **First author（year）** | **JBI score** | **Quality** | **1** | **2** | **3** | **4** | **5** | **6** | **7** | **8** | **9** | **-** |
| --- | --- | --- | --- | --- | --- | --- | --- | --- | --- | --- | --- | --- |
| Blüher, 2015 | 7 | High | Yes | Yes | Yes | Yes | Yes | Yes | No | Yes | Unclear | - |
| Brod, 2009 | 8 | High | Yes | Yes | Yes | Yes | Yes | Unclear | Yes | Yes | Yes | - |
| Gonz'alez-Saldivar, 2022 | 4 | Low | No | Unclear | Unclear | Yes | Yes | No | Unclear | Yes | Yes | - |
| Han, 2022 | 6 | Low | Yes | No | No | Yes | Yes | Yes | Unclear | Yes | Yes | - |
| Herzig, 2019 | 9 | High | Yes | Yes | Yes | Yes | Yes | Yes | Yes | Yes | Yes | - |
| Ishii, 2012 | 8 | High | Yes | Unclear | Yes | Yes | Yes | Yes | Yes | Yes | Yes | - |
| Ishii, 2018 | 6 | Low | Yes | Yes | Unclear | Unclear | Yes | Yes | Unclear | Yes | Yes | - |
| Morris, 2021 | 6 | Low | Yes | Yes | Yes | No | Yes | Yes | Unclear | Yes | No | - |
| Rogers, 2017 | 5 | Low | Yes | No | No | Yes | Yes | Unclear | Unclear | Yes | Yes | - |
| Sav, 2016 | 8 | High | Yes | Yes | Yes | No | Yes | Yes | Yes | Yes | Yes | - |
| Spencer-Bonilla, 2021 | 6 | Low | Yes | Yes | No | Yes | Yes | Unclear | Unclear | Yes | Yes | - |
| Vijan, 2005 | 6 | Low | Yes | Yes | Yes | Yes | Yes | Yes | No | No | No | - |
| NOTE: ITEMS OF CHECKLIST FOR PREVALENCE STUDIES  1. Was the sample frame appropriate to address the target population?  2. Were study participants sampled in an appropriate way?  3. Was the sample size adequate?  4. Were the study subjects and the setting described in detail?  5. Was the data analysis conducted with sufficient coverage of the identified sample?  6. Were valid methods used for the identification of the condition?  7. Was the condition measured in a standard, reliable way for all participants?  8. Was there appropriate statistical analysis?  9. Was the response rate adequate, and if not, was the low response rate managed appropriately? | | | | | | | | | | | | |
| **First author（year）** | **JBI score** | **Quality** | **1** | **2** | **3** | **4** | **5** | **6** | **7** | **8** | **9** | **10** |
| Bohlen, 2012 | 4 | Low | Unclear | Yes | Yes | Yes | Unclear | No | No | Unclear | Unclear | Yes |
| Bustillos, 2020 | 6 | Low | Unclear | Yes | Yes | Yes | Yes | No | No | Unclear | Yes | Yes |
| Cotugno, 2015 | 6 | Low | No | Yes | Yes | Yes | Yes | No | No | Unclear | Yes | Yes |
| Crutzen, 2021 | 7 | Low | Unclear | Yes | Yes | Yes | Unclear | Yes | Yes | Unclear | Yes | Yes |
| Dambha-miller, 2018 | 5 | Low | No | Yes | Unclear | Yes | Unclear | No | No | Yes | Yes | Yes |
| Espinoza, 2020 | 8 | High | Yes | Yes | Yes | Unclear | Yes | No | Yes | Yes | Yes | Yes |
| Fritschi, 2022 | 4 | Low | No | Yes | Yes | Yes | Yes | No | No | Unclear | Unclear | Unclear |
| Haider, 2021 | 6 | Low | No | Yes | Yes | Yes | Yes | No | No | No | Yes | Yes |
| Kristensen, 2018 | 8 | High | Yes | Yes | Yes | Yes | Yes | No | Unclear | Yes | Yes | Yes |
| Litterbach, 2020 | 4 | Low | Unclear | Unclear | Unclear | Yes | Yes | No | No | No | Yes | Yes |
| Mandrik, 2013 | 4 | Low | No | Yes | Yes | Unclear | Unclear | No | Yes | No | Unclear | Yes |
| Nair, 2007 | 5 | Low | Unclear | Yes | Yes | Yes | Yes | No | No | Unclear | Unclear | Yes |
| Spencer-Bonilla, 2021 | 4 | Low | No | Yes | Yes | Unclear | Unclear | No | Unclear | Unclear | Yes | Yes |
| Tanenbaum, 2016 | 6 | Low | Unclear | Yes | Yes | Yes | Yes | No | No | Unclear | Yes | Yes |
| Vijan, 2005 | 4 | Low | No | Yes | Yes | Unclear | Unclear | No | No | No | Yes | Yes |
| NOTE: ITEMS OF CHECKLIST FOR QUALITATIVE RESEARCH  1. Is there congruity between the stated philosophical perspective and the research methodology?  2. Is there congruity between the research methodology and the research question or objectives?  3. Is there congruity between the research methodology and the methods used to collect data?  4. Is there congruity between the research methodology and the representation and analysis of data?  5. Is there congruity between the research methodology and the interpretation of results?  6. Is there a statement locating the researcher culturally or theoretically?  7. Is the influence of the researcher on the research, and vice- versa, addressed?  8. Are participants, and their voices, adequately represented?  9. Is the research ethical according to current criteria or, for recent studies, and is there evidence of ethical approval by an appropriate body?  10. Do the conclusions drawn in the research report flow from the analysis, or interpretation, of the data? | | | | | | | | | | | | |

STable 3 Result of panel feedback

| **Subjects** | **Characteristics** | **F (Feasibility)** | **A (Appropriateness)** | **M (Meaningful)** | **E (Effectiveness)** | **TOTAL** |
| --- | --- | --- | --- | --- | --- | --- |
| **P1** | F, 76 yrs, Y3 | 4 | 3 | 4 | 3 | 14 |
| **P2** | F,72 yrs, Y3 | 4 | 3 | 4 | 3 | 14 |
| **P3** | M, 75 yrs, Y3 | 3 | 4 | 4 | 4 | 15 |
| **P4** | M, 25 yrs, Y1 | 4 | 4 | 4 | 3 | 15 |
| **HP1** | F, 32 yrs, Y1, E2 | 4 | 3 | 4 | 3 | 14 |
| **HP2** | M, 35 yrs, Y2, E2 | 4 | 3 | 4 | 4 | 15 |
| **HP3** | F, 24 yrs, Y1, E1 | 3 | 4 | 4 | 3 | 14 |
| **HP4** | F, 28 yrs, Y1, E2 | 4 | 3 | 4 | 3 | 14 |
| **CVI** |  | 0.94 | 0.84 | 1.00 | 0.81 | 0.90 |
| scores: 1=not relevant, 2=weakly relevant, 3=relevant, 4=strongly relevant | | | | | | |
| Gender, M= male, F= female;  Years in practice or durantion, Y1= <5 years in practice, Y2= 5-10 years in practice, Y3= >10 years in practice;  Educational background, E1= bachelor’s degree, E2= Master's degree, E3= PhD degree. | | | | | | |
